# Revealing the Mechanisms of Enhanced β-Farnesene Production in *Yarrowia lipolytica* through Metabolomics Analysis

**DOI:** 10.3390/ijms242417366

**Published:** 2023-12-11

**Authors:** Qianxi Liu, Haoran Bi, Kai Wang, Yang Zhang, Biqiang Chen, Huili Zhang, Meng Wang, Yunming Fang

**Affiliations:** National Energy R&D Center of Biorefinery, College of Life Science and Technology, Beijing University of Chemical Technology, Beijing 100029, China; 2022201232@buct.edu.cn (Q.L.); 2023700073@buct.edu.cn (K.W.); 2023700015@buct.edu.cn (Y.Z.); chenbq@mail.buct.edu.cn (B.C.); zhhl@mail.buct.edu.cn (H.Z.); fangym@mail.buct.edu.cn (Y.F.)

**Keywords:** metabolomics analysis, β-Farnesene, *Yarrowia lipolytica*, genetic engineering, fermentation optimization

## Abstract

β-Farnesene is an advanced molecule with promising applications in agriculture, the cosmetics industry, pharmaceuticals, and bioenergy. To supplement the shortcomings of rational design in the development of high-producing β-farnesene strains, a Metabolic Pathway Design-Fermentation Test-Metabolomic Analysis-Target Mining experimental cycle was designed. In this study, by over-adding 20 different amino acids/nucleobases to induce fluctuations in the production of β-farnesene, the changes in intracellular metabolites in the β-farnesene titer-increased group were analyzed using non-targeted metabolomics. Differential metabolites that were detected in each experimental group were selected, and their metabolic pathways were located. Based on these differential metabolites, targeted strain gene editing and culture medium optimization were performed. The overexpression of the coenzyme A synthesis-related gene pantothenate kinase (*PanK*) and the addition of four mixed water-soluble vitamins in the culture medium increased the β-farnesene titer in the shake flask to 1054.8 mg/L, a 48.5% increase from the initial strain. In the subsequent fed-batch fermentation, the β-farnesene titer further reached 24.6 g/L. This work demonstrates the tremendous application value of metabolomics analysis for the development of industrial recombinant strains and the optimization of fermentation conditions.

## 1. Introduction

β-Farnesene, an acyclic sesquiterpene compound, as an advanced molecule, finds extensive applications across a multitude of fields, including pharmaceuticals, cosmetics, and bioenergy [1,2]. The synthesis of Vitamin E with β-farnesene as the precursor is cost-effective, simple, and efficient, demonstrating substantial economic potential [3]. Moreover, β-farnesene, used as an intermediary in aviation fuel, contributes to bio-aviation fuel [4]. Compared to traditional aviation fuel, bio-aviation fuel can reduce greenhouse gas emissions by 50% to 90% over its full life-cycle, resulting in a markedly significant CO_2_ emission reduction effect [5]. The advancement of synthetic biology techniques has paved the way for the biosynthesis of β-farnesene in microorganisms using renewable resources, offering a viable alternative to traditional chemical synthesis or plant extraction processes [6,7]. This development undoubtedly injects vitality into the large-scale production of β-farnesene. Currently, the market share of farnesene produced through biosynthesis is nearly 100%. However, the scaled production of β-farnesene using microbial cell factories currently still confronts issues such as relatively low yield, suboptimal efficiency, and elevated production costs.

*Yarrowia lipolytica*, commonly known as oleaginous yeast, is renowned for its high TCA cycle flux and ability to hydrolyze lipids [8]. This yeast species exhibits a unique metabolic pathway that particularly excels in directing metabolic flow towards acetyl-CoA [8]. Furthermore, it demonstrates remarkable performance in high-density fermentation and environmental tolerance [9]. In the past decade, *Y. lipolytica* has drawn considerable attention in the field of industrial biotechnology due to its ability to produce high-value-added compounds [10]. These compounds have a wide range of applications, including biofuels, oleochemicals, and nutraceuticals [11,12,13,14]. Employing *Y. lipolytica* as a host for β-farnesene production has yielded surprisingly encouraging results. Owing to its broad substrate spectrum, *Y. lipolytica* is capable of employing various substrates such as sugars, lipids, and acetates for the synthesis of β-farnesene (Table 1) [14,15,16,17]. The metabolic flux and farnesene production can be maximized by strategically manipulating and optimizing the mevalonate (MVA) pathway. Strategies encompass engineering the rate-limiting enzymes in the MVA pathway, enhancing the metabolic intensity from acetyl-CoA to farnesene, optimizing the supply of cofactors, and adjusting the efficiency of metabolic pathways through strategies such as modular regulation and push-pull limitations [18,19,20]. Low concentrations of enzymes and substrates in the cytoplasm often limit the production rate of terpenoid compounds. Fusion of key enzymes can significantly enhance the titer of farnesene and reduce the accumulation of by-products [21,22]. The isopentenol utilization pathway (IUP) was designed for the production of IPP and DMAPP from isopentenol isomers. This artificial pathway, consisting of only two steps, consumes one ATP and is decoupled from central carbon metabolism, thus promoting increased carbon efficiency and farnesene production [23]. Despite the fact that the optimal titer of farnesene in *Saccharomyces cerevisiae* has reached up to 130 g/L [24], the natural MVA pathway is strictly regulated, but the mechanisms involved are not fully understood. Apart from a few colored terpenoid compounds, most terpenoids lack high-throughput screening methods. To acquire engineered strains with superior traits and production capabilities, more sophisticated metabolic pathway editing is necessary.

Existing genome-scale metabolic models indicate that *Y. lipolytica* encompasses over 1800 common metabolites, more than 2200 common reactions, and over 1000 significant genes [25]. Due to the lack of in-depth and comprehensive understanding of cellular metabolic mechanisms, relying solely on rational design of metabolic pathways often results in limited choices for host strain modification targets, thereby affecting the iteration efficiency of the engineered strains. In the process of genetically engineered strain development, the Metabolic Pathway Design-Fermentation Test-Metabolomic Analysis-Target Mining cycle is implemented. This method integrates strain design, genetic engineering modification, fermentation testing, and omics analysis to enhance strain performance and address production bottlenecks [26]. As an integral component within this framework, the tools of metabolomic analysis offer conditions conducive to the comprehensive exploration of intracellular metabolite variations [27,28]. Previous research has indicated that metabolomic analysis techniques can aid in regulating the global metabolic network of *Y. lipolytica*, understanding the molecular mechanisms of amino acid and lipid metabolism in oleaginous yeast, and improving lipid production [29,30].

**Figure 1:**
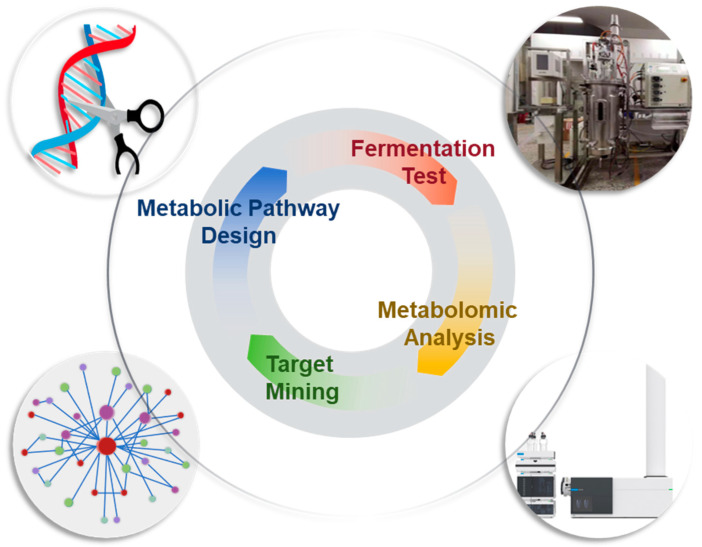
The schematic diagram of the experimental cycle constructed in this study.

Liquid Chromatography Quadrupole Time-of-Flight Mass Spectrometry (LC-QTOF-MS) is recognized as a non-targeted metabolomics analytical method capable of analyzing extracellular and intracellular metabolites. Owing to its broad dynamic range and high sensitivity, it is employed as an effective tool for characterizing a wide array of metabolites [31]. Previously, it has been extensively applied in various fields, such as observing metabolite variations in fermented food and beverages, identifying bioactive compounds, and conducting disease analysis [32,33,34,35]. In this study, initially, a set of amino acid/nucleobase addition experiments were designed during the β-farnesene fermentation process in *Y. lipolytica*, and differentiated cell samples were obtained based on the difference in β-farnesene titer. Subsequently, non-targeted metabolite analysis was performed using LC-QTOF-MS, and metabolic pathway localization was conducted based on the significantly differentiated compounds identified. The pantothenate synthesis pathway and the phospholipid synthesis pathway were identified as potential targets for enhancing β-farnesene synthesis. Ultimately, through the optimization of the fermentation medium and editing of the corresponding metabolic pathways, the titer of β-farnesene in *Y. lipolytica* was successfully improved and was validated during the fermentation process at a 30 L scale (Figure 1). This work provides new insights and feasible strategies for the development of terpene-synthesizing strains of *Y. lipolytica*.

## 2. Results and Discussion

### 2.1. The Impact of Amino Acids/Nucleobases Supplementation on β-Farnesene Biosynthesis

Studies have demonstrated that amino acids/nucleobases are among the critical factors influencing yeast fermentation. In the context of industrial applications, the control over the composition and concentration of amino acids is anticipated to aid in bolstering productivity and enhancing the value of fermentation products [36]. Nevertheless, few investigations have delved into the impact of amino acids on the production of the terpene compound β-farnesene via *Y. lipolytica* fermentation. In this research, 20 distinct amino acid/nucleobase aqueous solutions with a concentration of 10 mM were individually introduced into the fermentation medium. These amino acids/nucleobases included adenine, alanine, arginine, asparagine, aspartic acid, cysteine, glutamine, glutamic acid, glycine, leucine, lysine, methionine, phenylalanine, proline, serine, threonine, tryptophan, tyrosine, uracil, and valine. The influence of these singular amino acid/nucleobase supplements on the growth of *Y. lipolytica* and the synthesis of β-farnesene was examined.

**Figure 2:**
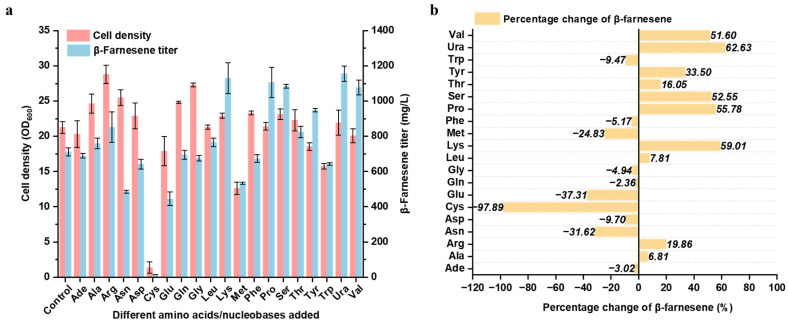
The effects of amino acids/nucleobases on β-Farnesene production by *Y. lipolytica*. (**a**) Cell density and β-farnesene titer; (**b**) Relative change rate, defined as the level of variation in β-farnesene titer in the medium supplemented with amino acids/nucleobases compared to that in the medium without amino acid/nucleobase supplementation. Strains were cultivated in shake flasks for 96 h with 10 mM amino acid/nucleobase added. Experiments were performed in triplicates.

As can be observed from Figure 2, the exogenous addition of most amino acids/nucleobases promoted the growth of the strain compared to the control group, with the contributions of arginine and glutamine being particularly notable. However, the addition of cysteine severely inhibited the growth of the strain (Figure 2a). The addition of certain amino acids visibly enhanced the titer of β-farnesene. The supplementation of valine, uracil, serine, proline, and lysine enabled the accumulation of β-farnesene to reach 1076.9 mg/L, 1155.3 mg/L, 1083.7 mg/L, 1106.6 mg/L, and 1129.5 mg/L respectively at the 96th hour of fermentation (Figure 2a), representing an increase of 51.6%, 62.6%, 52.6%, 55.8%, and 59.0% respectively compared to the yield without amino acid addition (Figure 2b). The experimental results suggest that the excessive addition of certain amino acids/nucleobases contributes to the accumulation of biomass and the synthesis of β-farnesene. This is consistent with a recent study that found that the addition of amino acids, particularly ketogenic amino acids, aids in the production of docosahexaenoic acid (DHA) in *Y. lipolytica*. For example, L-lysine, which can be stored and continuously degraded in vacuoles, thereby achieving long-term activation, contributes to the enhancement of DHA precursor synthesis [37]. Given the substantial fluctuations in β-farnesene titer and cell biomass, it is hypothesized that the addition of amino acids/nucleobases may have instigated disturbances in the intracellular metabolism of *Y. lipolytica* and the metabolic synthesis of β-farnesene. Considering that the excessive addition of amino acids/nucleobases may lead to a significant increase in production costs, and in an effort to identify effective metabolic targets that can enhance β-farnesene synthesis, intracellular metabolite detection analyses were conducted on cell samples from the amino acid/nucleobase supplementation groups (including valine, uracil, serine, proline, and lysine) where the β-farnesene titer increase ratio exceeded 50% (Figure 2b).

### 2.2. Metabolomics Analysis and Localization of Differential Metabolites

**Figure 3:**
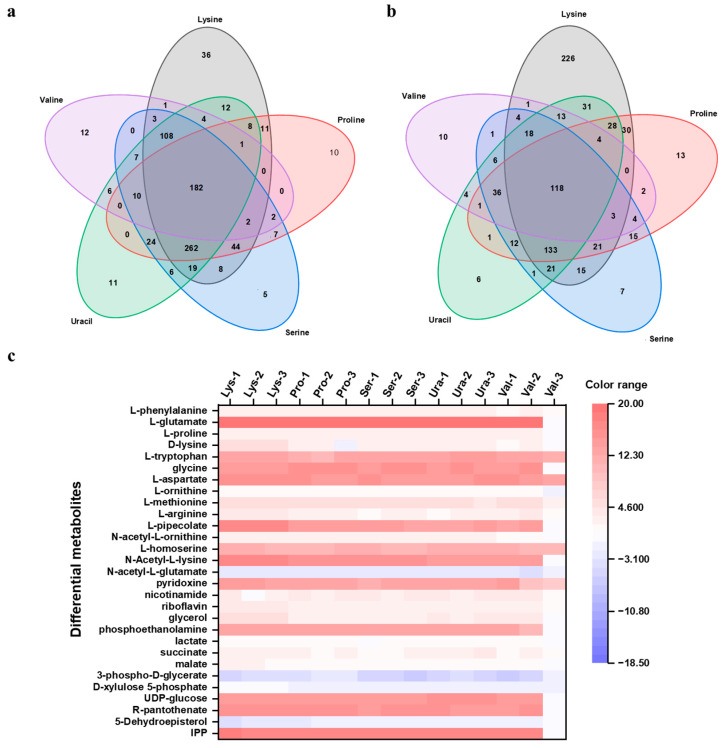
Results of intracellular metabolism analysis. Venn diagram of (**a**) upregulated and (**b**) downregulated differential metabolites appearing in all five experimental groups; (**c**) Heatmap of clustering analysis of differential metabolites (red indicates upregulated metabolites and blue indicates downregulated metabolites).

In an endeavor to investigate the metabolic implications induced by the aforementioned amino acids/nucleobase, which positively influence β-farnesene synthesis, intracellular metabolites were extracted from the strains during their vigorous growth phase (that is, at the 48th hour of fermentation) in the amino acid/nucleobase supplementation experiments. These metabolites were subsequently analyzed employing LC-QTOF-MS. The intracellular metabolite data were processed in accordance with the methods described in the Materials and Methods section (Section 3.5). The processed data were imported into Mass Profiler Professional Version 15.1 (MPP) provided by Agilent Technologies for subsequent multivariate data analysis (Section 3.5). Metabolites with an adjusted *p*-value ≤ 0.05 and an absolute fold change ≥ 2.0 were defined as differential metabolites. Specifically, metabolites with a fold change ≥ 2.0 were defined as upregulated, while those with a fold change ≤ −2.0 were considered downregulated (Figure S2). Upon statistical analysis, the quantities of upregulated and downregulated differential metabolites, as well as the total counts detected in the five experimental groups with added amino acids/nucleobase compared to the control group, are presented in Table 2. Due to the large number of differential metabolites, to narrow the scope and increase the precision of potential experiments, upregulated and downregulated differential metabolites that were consistently detected across all five experimental groups were selectively chosen. Among the selected metabolites, 182 upregulated differential metabolites and 118 downregulated differential metabolites were consistently detected across all five experimental groups (Figure 3a,b). Further, the metabolites screened at this stage were compared with an Agilent METLIN standard metabolite database for metabolite identification, and the minimum overall score was 70%. In cases where multiple compounds were matched, the compound with the highest score was selected.

Significant differences were observed at the metabolite level between the β-farnesene-enhanced production group and the control group without added amino acids/nucleobase. A heatmap clustering analysis was performed on differential metabolites that appeared in all experimental groups (Figure 3c). In this context, red indicates upregulation of metabolite levels, while blue indicates downregulation. These differential metabolites can mainly be classified into the following categories: amino acids, B vitamins, glycerophospholipids, central metabolites, and metabolites related to terpenoid compound synthesis.

The analysis results indicate that the addition of amino acids/nucleobase directly impacts intracellular amino acid metabolism, with higher levels of glutamate, tryptophan, glycine, glutamine, aspartate, and homocysteine. The accumulation of intracellular amino acids can not only occur through self-synthesis but also through transformation between different amino acids. For example, the metabolic network composed of L-arginine, L-proline, L-ornithine, and L-glutamine centered on L-glutamate becomes active under the condition of amino acid addition [38], increasing the cell’s resistance, promoting cell growth and production [36]. As the simplest amino acid, glycine is interconvertible with serine and couples with central carbon metabolism, improving cellular metabolic levels [39]. Tryptophan can be converted to the downstream active molecule NAD^+^, which can be phosphorylated to NADP^+^ by NAD^+^ kinase and reduced to NADH and NADPH [40], respectively. Given that the synthesis of terpenoid compounds requires the consumption of substantial quantities of the cofactor NAD(P)H [41], the upregulation of tryptophan may play a significant role in energy metabolism in β-farnesene synthesis. In addition to this, alterations in amino acid metabolism indirectly induced the conversion of intermediates in amino acid synthesis, where four types of intermediate metabolites were upregulated. These include L-pipecolic acid, a degradation metabolite of L-lysine; N-acetylornithine, an intermediate in the synthesis of amino acids such as arginine; L-homoserine, an intermediate in the synthesis of serine and methionine; and N-acetyl-L-lysine, a derivative of lysine. Conversely, N-acetyl-L-glutamate, a derivative of glutamic acid, was downregulated (Figure 3c).

In addition, it was observed that the intracellular responses of some B-vitamin metabolites increased in the experimental group with amino acid/nucleobase addition compared to the control group, including pyridoxine, niacin, riboflavin, and pantothenic acid. The B-vitamins are water-soluble compounds involved in cellular metabolism. They can stimulate yeast growth and are commonly added to yeast fermentation medium as micronutrients [42]. Pyridoxine, acting as a cofactor for numerous essential enzymes in the organism, participates in a variety of enzymatic reactions, such as amino acid metabolism and fatty acid metabolism [43]. An increase in riboflavin levels led to an enhancement in the intracellular content of FADH_2_, a reduced form of flavin adenine dinucleotide (FAD) and a carrier of protein-bound, reduced coenzyme involved in electron transfer and ATP production in the oxidative phosphorylation pathway [43]. This provides energy for the production of β-farnesene. The synthesis of β-farnesene initiates from the condensation of two acetyl-CoA molecules, with calcium pantothenate acting as a precursor for CoA synthesis [44]. An increase in its level promotes the synthesis of the CoA portion in acetyl-CoA, playing a key role in the enhancement of β-farnesene titer. Collectively, the mechanism and reasons for β-farnesene titer increase were explained and analyzed at the metabolite level.

### 2.3. Gene Editing and Culture Medium Optimization Based on Metabolomic Analysis

**Figure 4:**
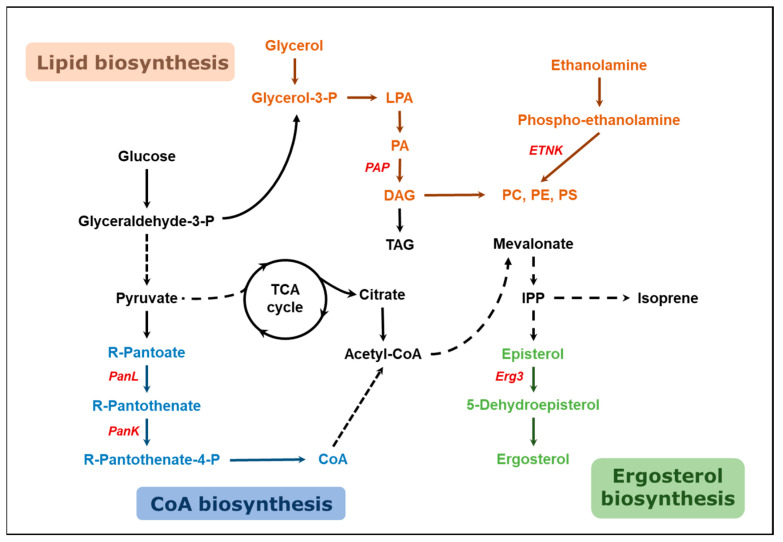
Schematic diagram of metabolic pathways involved in the identified differential metabolites. The blue text and arrows represent the CoA biosynthesis pathway, the green text and arrows represent the ergosterol biosynthesis pathway, the orange text and arrows represent the lipid biosynthesis pathway, and the selected target genes for modification are indicated in red text. PanL, pantoate-β-alanine ligase; PanK, pantothenate kinase; LPA, lysophosphatidic acid; PA, phosphatidic acid; PAP, phosphatidic acid phosphohydrolase; DAG, diacylglycerol; TAG, triacylglycerol; ETNK, Ethanolamine Kinase; PC, phosphatidylcholine; PE, phosphatidylethanolamine; PS, phosphatidylserine; IPP, isopentenyl diphosphate; Erg3, C-5 sterol desaturase.

It has been proven that the excess addition of certain types of amino acids/nucleobase has a positive effect on the growth of *Y. lipolytica* and the production of β-farnesene. However, considering the cost pressure of adding a large amount of amino acids/nucleobases in large-scale fermentation, it is hoped to explore new changes at the metabolite level and reproduce them using genetic engineering. In an in-depth exploration of the inherent causes for the observed increase in β-farnesene production, analysis was carried out on the Kyoto Encyclopedia of Genes and Genomes (KEGG) to assess the distribution of differential metabolites in metabolic pathways, with analysis conducted from both the metabolic pathway and genetic perspectives. Emphasis was placed on 5-Dehydroepisterol, which displayed a downregulation, and three key differentiating metabolites that were upregulated: R-pantothenate, glycerol, and phosphoethanolamine. These metabolites are implicated in the ergosterol synthesis pathway, the pantothenic acid and coenzyme A synthesis pathway, the triacylglycerol synthesis pathway, and the phospholipid synthesis pathway, respectively (Figure 4).

The synthesis of coenzyme A (CoA) initiates with pantothenate, which is converted to pantetheine under the action of pantothenate-β-alanine ligase (PanL). Pantetheine is then phosphorylated by pantothenate kinase (PanK) to form 4′-phosphopantetheine. Following the condensation of 4′-phosphopantetheine with cysteine, a decarboxylation reaction takes place to produce 4′-phospho-pantetheine. The addition of the 5′-AMP part of ATP forms dephospho-CoA, which subsequently undergoes phosphorylation at the 3′-hydroxyl to produce CoA [45]. PanL and PanK are key enzymes that regulate this pathway and are crucial for controlling the intracellular content of CoA [46]. The study considers enhancing the intracellular coenzyme A synthesis pathway through the overexpression of *PanL* and *PanK*. The aim is to augment the accumulation of acetyl-CoA to foster the production of β-farnesene. Phosphoethanolamine serves as a precursor for phosphatidylcholine and phosphatidylethanolamine, participating in the synthesis and renewal of cell membranes. Ethanolamine kinase (ETNK) is responsible for the phosphorylation of ethanolamine to phosphoethanolamine [47]. By enhancing the intracellular expression of ETNK, the concentration of phosphoethanolamine is increased, leading to alterations in the structural characteristics of the cell membrane. This influences the folding and activity of several membrane proteins, thereby affecting the excretion efficiency of the product β-farnesene [48]. Glycerol is converted to phosphatidic acid through a series of intracellular reactions. Under the action of phosphatidic acid phosphatase (PAP), it is transformed into diacylglycerol (DAG), which then synthesizes key compounds impacting membrane structural features, such as phosphatidylcholine (PC) and phosphatidylethanolamine (PE) [49]. Furthermore, ergosterol is a critical component of eukaryotic cell membranes, maintaining the fluidity, permeability, and activity of membrane-associated proteins. 5-Dehydroepisterol occupies a pivotal position in the synthetic metabolic pathway of ergosterol [50]. Here, PAP and C-5 sterol desaturase (ERG3) were selected as potential targets, and strategies were implemented for their respective knockout and overexpression.

**Figure 5:**
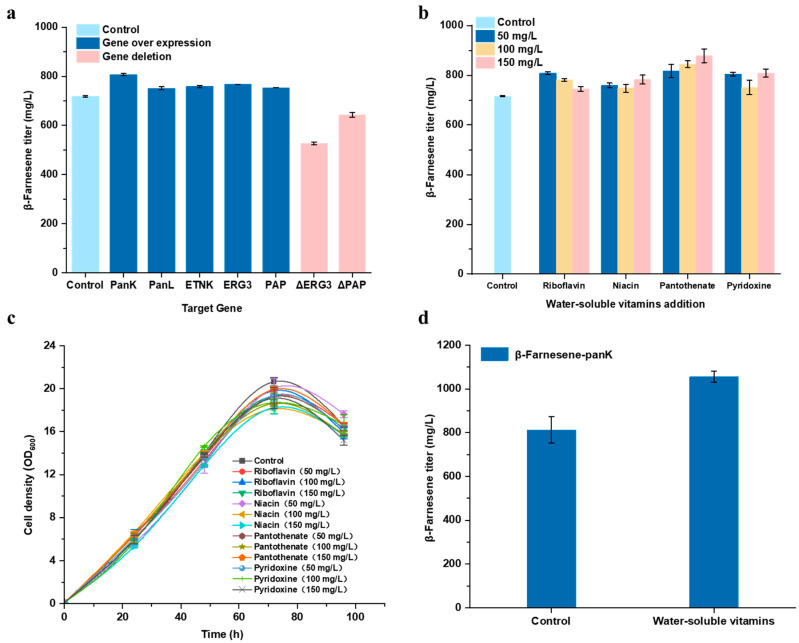
(**a**) Production of β-farnesene with different genetic modification strategies; The effects of water-soluble vitamins on (**b**) β-farnesene production and (**c**) cell growth; (**d**) The β-farnesene titer of the recombinant strain in the medium supplemented with a mixture of water-soluble vitamins. Strains were cultivated in shake flasks for 96 h, and experiments were performed in triplicates.

The results indicate that compared to the control group, the strains overexpressing the aforementioned genes (*PanK*, *PanL*, *ETNK*, *ERG3*, and *PAP*) demonstrated varying degrees of enhancements in the titer of β-farnesene. The most significant effect was observed in the strain overexpressing *PanK*, where the β-farnesene titer accumulated to 807.6 mg/L, an increase of 12.5% (Figure 5a). This validates that enhancing the intracellular coenzyme A synthesis pathway and altering cell membrane structural characteristics can promote the synthesis of β-farnesene. Conversely, strains with *ERG3* and *PAP* knockouts exhibited a substantial reduction in the concentration of β-farnesene produced, indirectly confirming that altering cell membrane permeability can direct more β-farnesene toward the exterior of the cell.

In Section 2.2, the differential metabolites identified, namely pyridoxine, niacin, riboflavin, and pantothenate, showed increased levels compared to the control group without added amino acids/nucleobases. In conjunction with the enhanced titer of β-farnesene, the addition of certain amino acids/nucleobase indirectly led to the accumulation of these water-soluble vitamins within the cell, thereby promoting the synthesis of β-farnesene. Based on these metabolite detection results, fermentation conditions were adjusted by increasing the exogenous supply of water-soluble vitamins (including pyridoxine, niacin, riboflavin, and calcium pantothenate) to the fermentation medium in hopes of boosting the titer of β-farnesene. The above-mentioned four types of water-soluble vitamins were separately added to the basic fermentation medium. Each water-soluble vitamin was added in three different concentrations: 50 mg/L, 100 mg/L, and 150 mg/L. Experiments were conducted to investigate the impact of these concentrations on the biosynthesis of β-farnesene. The results reveal (Figure 5b,c) that the growth of the strain was somewhat influenced after the addition of these water-soluble vitamins, with a slight decline in cell density. This evidence indicates that the exogenous addition of these compounds exerts a mild inhibitory impact on the normal growth of the strain, but it is within acceptable parameters. Moreover, the addition of different water-soluble vitamins to the medium resulted in a range of enhancements in the titer of β-farnesene, suggesting that the exogenous addition of water-soluble vitamins positively influences the synthesis and accumulation of β-farnesene. Considering the concentration gradient, as the added concentration increases, the titer of β-farnesene in the riboflavin group declines. This suggests that an excessively high concentration of riboflavin is actually unfavorable for the synthesis of β-farnesene. Among the elements, calcium pantothenate exhibited the most pronounced promoting effect on product synthesis. Within a reasonable concentration range, its promoting effect escalates as the added concentration increases. The experiment group, with the addition of 150 mg/L of calcium pantothenate, achieved a β-farnesene titer of 878.2 mg/L, an increase of 22.7%.

To further combine the preliminary experimental results, the *PanK* overexpressing strain was used as the research object, and mixed water-soluble vitamins (50 mg/L riboflavin, 150 mg/L niacin, 150 mg/L calcium pantothenate, and 50 mg/L pyridoxine) were added to the fermentation medium. As shown in Figure 5d, the experiment group supplemented with mixed water-soluble vitamins produced 29.9% more β-farnesene than the control group, reaching 1054.8 mg/L at 96 h. Through the excavation of differential metabolites and subsequent pathway localization, coupled with the utilization of genetic engineering techniques and optimized fermentation additives, we have essentially replicated the β-farnesene production levels observed when excessive amino acids/nucleobases were added, this titer represents a 48.5% increase compared to that of the initial strain under original fermentation conditions.

### 2.4. Fed-Batch Fermentation in a 30-Liter Fermenter

**Figure 6:**
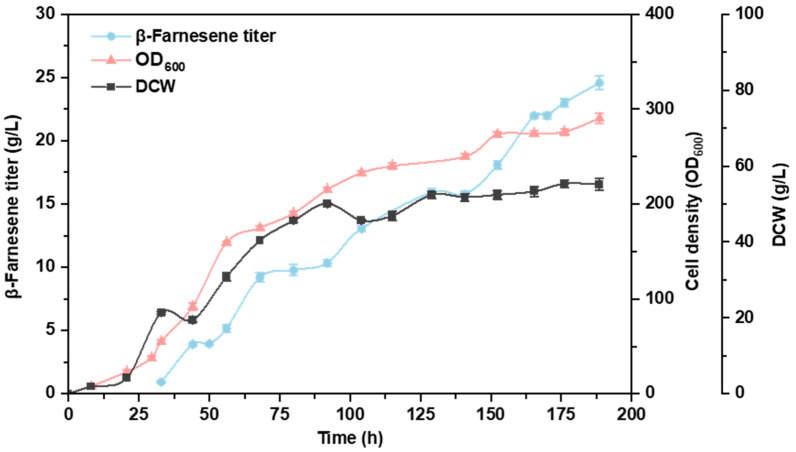
Results of the 30 L fed-batch fermentation. The duration of the fermentation process was 187 h, and the fermentation medium was supplemented with an optimized mixture of water-soluble vitamins.

To further investigate the fermentation performance of the recombinant strain in the optimized medium, fed-batch fermentation was conducted in a 30 L fermenter. The fermentation process lasted for 187 h. By the end of fermentation, the peak titer of β-farnesene was 24.6 g/L. During the fermentation process, the cell growth state was good, with a peak OD_600_ of 290 and a peak cell dry weight (DCW) of 55.3 g/L (Figure 6). It should be noted that, upon comparative analysis with our previous fermentation results, it was observed that the oleaginous yeast, *Y. lipolytica*, exhibits a high oxygen demand during the fermentation process. This factor appears to impose certain limitations on cell growth. Potential improvements in cellular oxygen uptake may be achieved through the optimization of bioreactor design and fermentation parameters [51]. Additionally, the introduction of hemoglobin into *Y. lipolytica* could potentially offer a strategy for enhancing terpene synthesis in large-scale fermentation, given its role in oxygen transport [52].

## 3. Materials and Methods

### 3.1. Plasmids and Strains Construction

The plasmid Prsf-3HA is used for gene knockout, which includes the ura3 expression cassette and the flanking 3HA sequences for selection marker recycling [53]. The plasmid pMO-ura is a free plasmid of *Y. lipolytica*, used for gene expression. Both of these were obtained from laboratory stock. DNA fragments were amplified using DNA polymerase (PrimeSTAR Max, Takara Bio, Beijing, China), and a seamless cloning kit (pEASY-Basic, TransGen Biotech, Beijing, China) was used for DNA fragment ligation and plasmid assembly. The plasmids, strains, and primers constructed and used in this study are listed in Tables S1–S3. The initial strain used was AYL119, a β-farnesene-producing strain of *Y. lipolytica* previously constructed in our lab [14]. A Frozen-EZ Yeast Transformation II Kit (Zymol Research, Irvine, CA, USA) was used for the preparation of competent cells and the construction of recombinant strains.

### 3.2. Shake Flask Fermentation

The shake-flask experiments were performed in 100 mL shake flasks containing 30 mL of SC-Glucose medium (30 g/L glucose, 5 g/L ammonium sulfate, and 1.7 g/L yeast nitrogen base). The culture medium was inoculated with an initial OD_600_ of 0.1, and under sterile conditions, 3 mL and 10 mM of 20 different amino acid/nucleobase solutions were added to the culture medium, respectively. A control group was replaced with an equivalent volume of sterile water. Cultivation was carried out at 30 °C and 300 rpm for 96 h. At 60 h, 15% (*v*/*v*) of n-nonane was added for β-farnesene extraction. The β-farnesene content in the organic layer was determined using the method described previously [14]. Experiments were performed in triplicates. 

### 3.3. Fed-Batch Fermentation

The fed-batch fermentation was performed in a 30 L fermenter (B. Braun Biotech International, Berlin, Germany); the initial working volume was 10 L, and the initial inoculation amount was 0.2 (OD_600_). The initial fermentation medium was the same as that in the shake flask, with the addition of an optimized water-soluble vitamin mixture. Fermentations were performed at 30 °C with pH 6.0 and an initial aeration rate of 1 vvm. The initial fermentation speed was 400 rpm, which was increased to 600 rpm after 36 h. A concentrated glucose solution (600 g/L) mixed with water-soluble vitamins was used for the supplement. Solutions of hydrochloric acid and potassium hydroxide were used to maintain the pH. Alpha-olefin was used to cover the fermentation broth for the extraction of β-farnesene.

### 3.4. Metabolite Extraction and LC-QTOF-MS Detection

Cells were collected at 48 h of fermentation, with cell counts ranging between 10^6^–10^7^. Normalization of various experimental groups was accomplished utilizing an OD_600_ measurement. Following a 2 min centrifugation at 12,000 rpm, the supernatant medium was discarded. The cells were then rinsed twice with 1 mL of ultrapure water to mitigate the interference from medium components. The samples were subsequently allowed to rest at −20 °C for 30 min before being lyophilized into a powder using a vacuum freeze-dryer. A volume of 150 μL of a 50% acetonitrile aqueous solution was then added to reconstitute the samples. To promote the complete dissolution of the metabolites, the samples were subjected to a 10 min ultrasonication in an ice-water bath at 4 °C, followed by a 15 min centrifugation at 12,000 rpm. Post dilution by a factor of ten, the supernatant was analyzed using an LC-QTOF-MS (1290 Infinity II UHPLC-6546 Q TOF LCMS system, Agilent Technologies Inc., Singapore, Singapore) system equipped with an InfinityLab Poroshell 120 HILIC-Z column (2.1 × 150 mm, 2.7 μm, PEEK-lined).

The chromatographic conditions are as follows. Flow rate: 0.3 mL/min; column temperature: 40 °C; injection volume: 2 μL; mobile phase A: Aqueous solution containing 15 mM ammonium acetate, 0.3% ammonia water, and 0.1% deactivator (InfinityLab Deactivator Additive); Mobile phase B: 90% acetonitrile solution containing 15 mM ammonium acetate, 0.3% ammonia water, and 0.1% deactivator (InfinityLab Deactivator Additive). The settings for the binary pump are shown in Table 3. The mass spectrometry conditions are as follows. Ion source: electrospray ion source; gas temperature: 325 °C; dryer flow rate: 6 L/min; spray air pressure: 35 psi (145 psi = 10^6^ Pa); capillary voltage of ion source: 4000 V; nozzle pressure: 1000 V; MS-TOF capillary voltage: 175 V; primary mass spectrum mass scan range (*m*/*z*): 50–1500; negative ion mode (ESI-) reference ion (*m*/*z*): 112.9855, 1033.9881; positive ion mode (ESI+) reference ion (*m*/*z*): 121.050873, 149.02332, 322.048121, 922.009798, 1221.990637, 1521.971475, 2421.91399; mode: MS; acquisition rate: 1.5 spectra/s; acquisition time: 666.7 ms/spectrum.

### 3.5. LC-MS Data Processing

The extraction of compound data was executed employing Mass Hunter Profinder Version 10.0 software provided by Agilent Technologies (Santa Clara, CA, USA), as delineated in prior work [54]. Specifically, a recursive Profinder workflow was engaged to derive compound data from all samples based on abundance profiles across *m*/*z* and retention time (RT) dimensions. The extraction process for the aqueous positive mode samples was conducted as follows: An RT extraction range of 0 to 14.7 min was employed, with a noise peak height filter set at ≥300 counts. Ion species included H^+^, Na^+^, K^+^, and a maximum charge state of 1. The alignment tolerance for RT was determined to be 0% + 0.2 min with a mass of 20 ppm + 2 mDa. ‘Find by Molecule Feature’ (MFE) parameters were put into operation with a score ≥ 65, and ‘Find by Ion’ (FbI) parameters were implemented with a score ≥ 50. The extraction parameters for the aqueous negative mode samples mirrored those of the positive mode, with the exception of the ion species, which was -H. Here, too, the MFE parameters adopted had a score ≥ 65, and the FbI parameters utilized had a score ≥ 50.

The processed data was uploaded and subsequently imported into Mass Profiler Professional Version 15.1 (MPP), provided by Agilent Technologies for multivariate data analysis. Prior to statistical analysis, the abundance of all sample compounds was calibrated based on the average abundance of compounds in the control group, and a quality control filter was used to help eliminate compounds that interfere with the analysis. Subsequent multivariate data analysis included principal component analysis (PCA), *t*-test, and volcano plot (Figure S1). The samples compared in this study were independently and unrelatedly treated, so an unpaired *t*-test was used to calculate the *p*-values for normally distributed samples. The type of *p*-value calculation used an asymptotic algorithm, and multiple testing correction was performed through Benjamin Hochberg FDR to reduce the occurrence of false-negative results. The results of the *t*-test were presented in the form of a volcano plot, displaying compounds filtered by a combination of *t*-test (*p*-value ≤ 0.05) and fold change (fold change ≥ 2). These were identified as differential metabolites.

## 4. Conclusions

In conclusion, under the guidance of metabolomics analysis, modifications were undertaken on the identified CoA synthesis-related genes, and the addition of four water-soluble vitamins in the culture medium was optimized. This led to a successful increase of 48.5% in the titer of β-farnesene in shake-flask cultivation. Furthermore, in the fermentation scale-up process of 30 L, a β-farnesene production of 24.6 g/L was achieved. The results of metabolic analysis can provide scientific and targeted guidance for the optimization of fermentation conditions, thereby enhancing the titer and value of fermentation products. This work validates the practical significance of metabolic profiling in the regulation of fermentation strategies and strain development and also sets a precedent for future scale-up attempts, potentially paving the way for industrial applications.

## Figures and Tables

**Table 1:** β-Farnesene production titers, yields using specific substrates in *Y. lipolytica*.

Substrate	Fermentation Scale	Titer (g/L)	Yield (g/g)	Reference
Glucose	2-L fermenter	28.9	0.0855	[14]
Lignocellulosic hydrolysate	2-L fermenter	7.38	0.075	[15]
Oleic acid	5-L fermenter	35.2	0.170	[16]
Acetate	5-L fermenter	14.8	0.0558	[17]

**Table 2:** The quantities of differential metabolites in each experimental group.

	Lysine	Proline	Serine	Uracil	Valine
Upregulated	701	563	689	660	340
Downregulated	666	423	415	435	227
Total	1367	986	1104	1095	567

**Table 3:** Binary pump parameters.

Time (Min)	Pump A (%)	Pump B (%)	Flow (mL/Min)	Max Pressure Limit (Bar)
0.00	3.00	97.00	0.30	600.00
1.00	3.00	97.00	0.30	600.00
10.00	15.00	85.00	0.30	600.00
15.00	28.00	72.00	0.30	600.00
20.00	60.00	40.00	0.30	600.00
25.00	60.00	40.00	0.30	600.00

## Data Availability

The data presented in this study are available in the article and Supplementary Materials.

## Supplementary Materials

The following supporting information can be downloaded at https://www.mdpi.com/article/10.3390/ijms242417366/s1.
