# Supplementary material for: Revealing the Mechanisms of Enhanced β-Farnesene Production in Yarrowia lipolytica through Metabolomics Analysis
Source: Int J Mol Sci. 2023 Dec 11;24(24):17366. doi: 10.3390/ijms242417366 (PMC10743872; doi:10.3390/ijms242417366)
Supplement: Supplementary file 1 [file ijms-24-17366-s001.zip › ijms-2738810-supplementary.pdf]

Supplementary Materials for:

Revealing the Mechanisms of Enhanced β-Farnesene Production in *Yarrowia lipolytica* through Metabolomics Analysis

Qianxi Liu[1], Haoran Bi[1]*, Kai Wang[1], Yang Zhang[1], Biqiang Chen[1], Huili Zhang[1], Meng Wang[1*], Yunming Fang[1]

Author affiliations

[1] National Energy R&D Center of Biorefinery, College of Life Science and Technology, Beijing University of Chemical Technology, Beijing 100029, P.R. China

* Correspondence: Author to whom correspondence should be addressed.

Supplementary Figures

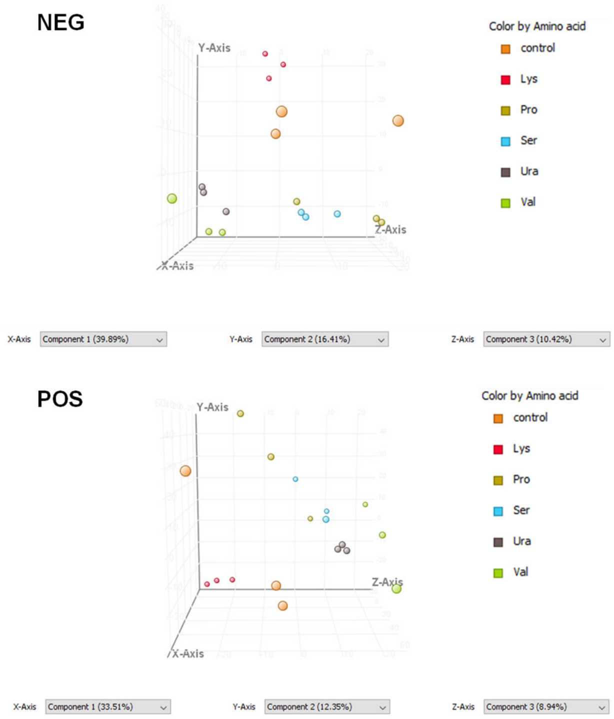

Figure S1. 3D PCA plot of different experimental groups in positive and negative polarity.

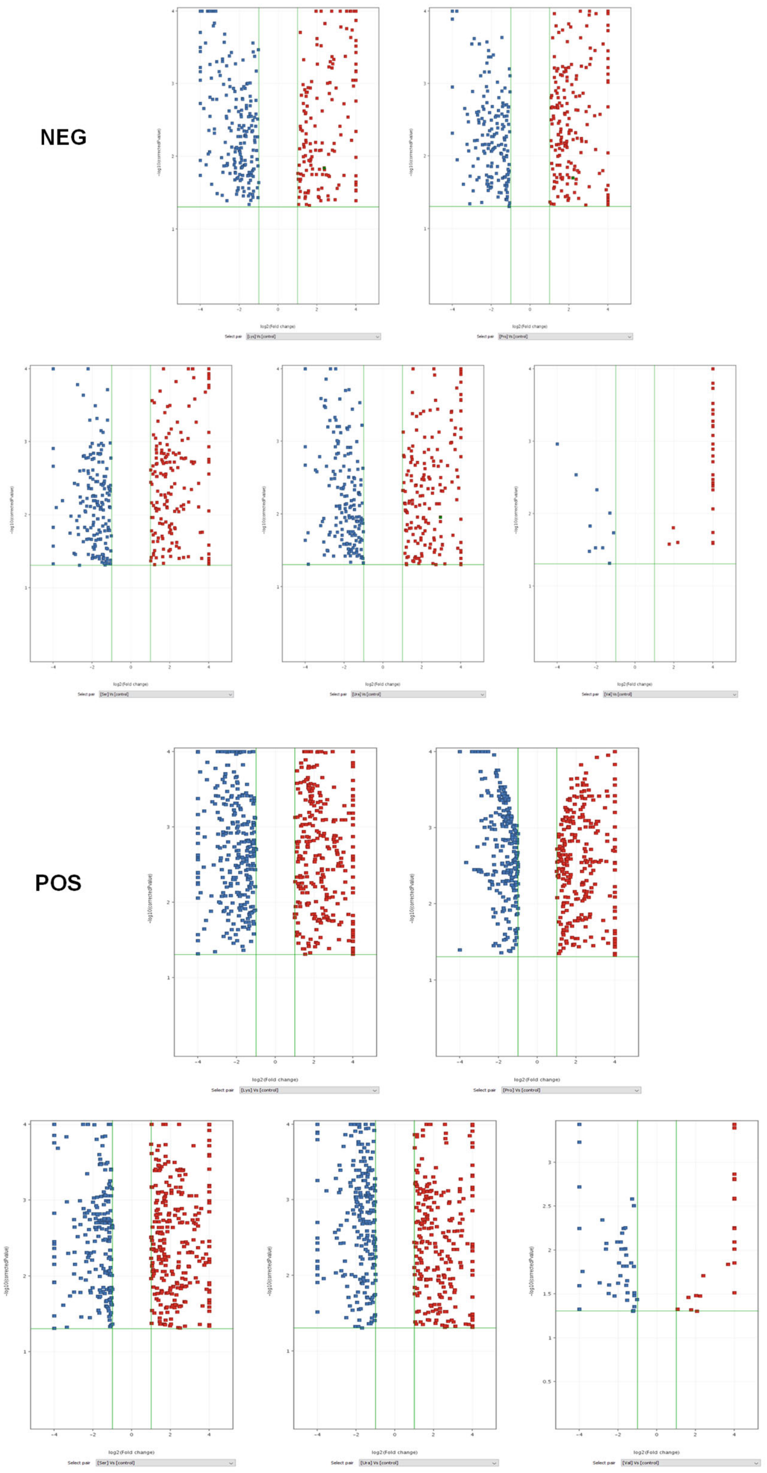

Figure S2. Volcano plot of metabolites under different amino acid/nucleobase additions in positive and negative polarity.

Supplementary Tables

Table S1. Strains used in this study.

Strains	Genotype or characteristic	Sources
E. coli	Trans10	TransGen
AYL119	AYL101, pMO-P_{TEFin}-*mmACL*-T_{xpr2}-P_{TEFin}-*ylAMPD*-T_{xpr2}-T_{xpr2}-P_{TEFin}-*ylYHM2*-T_{xpr2}	[14]
AYL119-1	AYL119, pMO-P_{TEFin}-*ylPanK*-T_{lip2}	This study
AYL119-2	AYL119, pMO-P_{TEFin}-*ylPanL*-T_{lip2}	This study
AYL119-3	AYL119, pMO-P_{TEFin}-*ylETNK*-T_{lip2}	This study
AYL119-4	AYL119, pMO-P_{TEFin}-*ylPAP*-T_{lip2}	This study
AYL119-5	AYL119, pMO-P_{TEFin}-*ylERG3*-T_{lip2}	This study
AYL119-6	AYL119, Δ*ylPAP*::3HA	This study
AYL119-7	AYL119, Δ*ylERG3*::3HA	This study

Table S2. Plasmids used in this study.

**Plasmids**	**Description**	**Sources**
pMO	Amp, mtOri, *URA3* marker, TEFin promoter and XPR2 terminator	Laboratory storage
pRSF	Kan, 3HA-*URA3* marker-3HA, TEFin promoter and XPR2 terminator	Laboratory storage
pMO-ylPanK	P_{TEFin}-*ylPanK*-T_{lip2}	This study
pMO-ylPanL	P_{TEFin}-*ylPanL*-T_{lip2}	This study
pMO-ylETNK	P_{TEFin}-*ylETNK*-T_{lip2}	This study
pMO-ylPAP	P_{TEFin}-*ylPAP*-T_{lip2}	This study
pMO-ylERG3	P_{TEFin}-*ylERG3*-T_{lip2}	This study
pRSF- ylPAP	*PAPup-3HA-URA3-3HA-PAPdown*	This study
pRSF- ylERG3	*ERG3up-3HA-URA3-3HA-ERG3down*	This study

Table S3. List of primers used in this study.

Names	Sequences (5’ > 3’)
pMOvec-F	GCTATTTATCACTCTTTACAACTTCTACCTCAACTATC
pMOvec-R	CTGCGGTTAGTACTGCAAAAAGTGCTG
ylPanK-F	CTTTTTGCAGTACTAACCGCAGATGCAACAAGCAACACAGGAACTG
ylPanK-R	GTTGTAAAGAGTGATAAATAGCCTACTGCATGAAACGCTCCAACTCC
ylPanL-F	CTTTTTGCAGTACTAACCGCAGATGTTGCGACCGGTGATTCG
ylPanL-R	GTTGTAAAGAGTGATAAATAGCTTAACACAGAACATTGTCAATGATTCTAGTCT
ylETNK-F	CTTTTTGCAGTACTAACCGCAGATGTCGCAACCAACATACCCAATG
ylETNK-R	GTTGTAAAGAGTGATAAATAGCTTACTTTGCCTCTCTTCCCTTCTTCC
ylPAP-F	CTTTTTGCAGTACTAACCGCAGATGTTGTCTTCCAGCTCCACCCT
ylPAP-R	GTTGTAAAGAGTGATAAATAGCTTAAACCTGGTTCTCGAGCTGAACATCG
ylERG3-F	CTTTTTGCAGTACTAACCGCAGATGGATATCGCTCTGGAGACCATCG
ylERG3-R	GTTGTAAAGAGTGATAAATAGCTTAATCCTGCTTGGTGTTTCGCTTGACA
ylERG3-UP-F	TTGCGTTGCGCCAATCTATACACTAGCCAGATGGCTTCTTTG
ylERG3-UP-R	CGTTTTACAACGGCGGCAGGTGTGTGTGTC
ylERG3-DM-F	TCTCTGTCTGCCATCTTTTGACCATGAAGCGAAGGACAAG
ylERG3-DM-R	ATGCCTGCCATGATGATACACGAGAGAGAGAGATAGC
pRSF-ERG3vec-F	CGTGTATCATCATGGCAGGCATTTGAGAAGCACACGGTC
pRSF-ERG3vec-R	GTGTATAGATTGGCGCAACGCAATTAATGTAAGTTAGCTCA
ylERG3-3HA-F	CCTGCCGCCGTTGTAAAACGACGGCCAGTCGAAC
ylERG3-3HA-R	TGGTCAAAAGATGGCAGACAGAGAGGTGAAGAAGAGG

ylPAP-UP-F	GTTGCGCCTGGGGCAAGAAGTTTCCTGGC
ylPAP-UP-R	CGTTTTACAACTTTGGGTGCGGCAAAGTTGATTTTCAG
ylPAP-DM-F	CTCTGTCTGCCACCAAACCAAACAATATAAAAACGGATCTGTAG
ylPAP-DM-R	CAAATGCCTGAGAGGGTCCTCCTTGAGGCAG
pRSF-PAPvec-F	GGAGGACCCTCTCAGGCATTTGAGAAGCACACGG
pRSF-PAPvec-R	CTTCTTGCCCCAGGCGCAACGCAATTAATGTAAGTTAGCTCAC
ylPAP-3HA-F	GCCGCACCCAAAGTTGTAAAACGACGGCCAGTCGAAC
ylPAP-3HA-R	TTGGTTTGGTGGCAGACAGAGAGGTGAAGAAGAGG

12

References

14. Bi, H.; Xu, C.; Bao, Y.; Zhang, C.; Wang, K.; Zhang, Y.; Wang, M.; Chen, B.; Fang, Y.; Tan, T. Enhancing precursor supply and modulating metabolism to achieve high-level production of β-farnesene in Yarrowia lipolytica. *Bioresour. Technol.* **2023**, *382*, 129171.
